# Editorial: Methods and applications in Psychology for Clinical Settings

**DOI:** 10.3389/fpsyg.2023.1192774

**Published:** 2023-06-14

**Authors:** Tindara Caprì, Casandra Isabel Montoro, Carmen María Galvez-Sánchez

**Affiliations:** ^1^Department of Life and Health Sciences, and Health Professions, Link Campus University, Rome, Italy; ^2^Department of Psychology, University of Jaén, Jaén, Spain

**Keywords:** Psychology for Clinical Settings, experimental techniques, technology, methods, general psychology

The main aim of this Research Topic was to highlight the latest experimental techniques and methods used to investigate relevant questions in Psychology for Clinical Settings. This Research Topic includes 11 papers, six original research articles, two opinion articles, one mini review, and one brief research report, which include recent techniques and up-to-date methods that contribute to advancing the science of Psychology for Clinical Settings.

The first study (Jerković et al.) examined the factor structure, internal consistency, and correlates of the Croatian version of the Hospital Anxiety and Depression Scale (HADS) for patients with multiple sclerosis (MS). A total of 179 patients with MS and 999 healthy subjects completed the HADS. The results of this study demonstrated that the HADS is a reliable and valid self-assessment scale and suggested that it be used in the clinical monitoring of the psychiatric and psychological status of patients with MS.

The second study (Cobo et al.) investigated whether motor anticipation in handwriting can be an indicator of motor dysfunction in schizophrenia. A total of 24 subjects with a diagnosis of schizophrenia and 24 healthy subjects performed an easy and brief handwriting task. The authors used three measures to evaluate motor anticipation: the time per stroke (duration), the path of the pen for each stroke (trajectory), and the number of velocity peaks (disfluency). The results indicated that patients with schizophrenia did not exhibit any signs of motor anticipation. This study supported the idea of using handwriting analysis as a quantitative, objective, and reliable tool to detect motor alterations in schizophrenia.

Two studies included in this Research Topic described the effects of two different therapeutic techniques used during the COVID-19 pandemic. The first study (Zwilling et al.) examined the wellbeing level of mothers of girls and women with Rett Syndrome (RTT) who were involved in a home-based, remotely supervised motor rehabilitation program before and during the Italian COVID-19 lockdown. In total, 40 subjects with RTT were randomly assigned to two groups: Group 1 received the intervention immediately before the lockdown, and Group 2 received it during the lockdown. The motor rehabilitation program consisted of an individualized daily physical activity program carried out for 12 weeks by the participants' parents and supervised every two weeks through Skype calls to plan, monitor, and accommodate individual activities to the participants' home lives. The results showed that the participants' mothers' wellbeing was similar in the two groups, indicating that the lockdown influenced the effect of the rehabilitation program. Thus, this study proposed that the motor intervention helped the mothers of patients with RTT to manage the new daily routine at home.

The second study, by Tao et al., described a new therapeutic technique to reduce psychological symptoms during the COVID-19 pandemic in China. This technique was the “Moving to Emptiness Technique” (MET), which combined traditional Chinese culture with relaxation and the operational process of Cognitive Behavioral Therapy (CBT). A total of 17 therapists treated 107 subjects using the MET. The participants were subdivided into two groups: a high-frequency symptom group and a low-frequency symptom group. The results showed that symptoms decreased significantly in both groups after the intervention, indicating that MET is a good therapeutic technique. However, the authors suggested caution in the interpretation of their results due to the small sample size.

Another study by Huang et al. examined the effects of hospital culture, self-efficacy, and achievement motivation on healthcare workers' perceived delivery of patient-centered care (PCC). In total, 1,612 healthcare workers from different levels of public hospitals completed a survey interview. The results suggested that self-efficacy, achievement motivation, and hospital culture were necessary to promote the use of PCC among healthcare workers.

The last research article (Ho et al.) included in this Research Topic investigated the effects of virtual reality nature experiences on the psychological and physiological stress of furniture factory employees. A total of 35 factory workers were assigned to two groups: an experimental group and a comparison group. The experimental group received virtual reality experiences consisting of 30-min nature-based 360° videos played in a headset. The intervention was conducted once a week for 12 weeks. The comparison group received no intervention; participants in this group freely performed their activities during their afternoon break. The results indicated that the experimental group showed an improvement in distress, depression, and anxiety compared to the comparison group.

With reference to two opinion articles included in the present Research Topic, the first by Metcalfe discussed practical considerations for behavioral health researchers when using open science. The author encouraged researchers to consider open science practices for their projects in pediatric medicine because they can be a viable tool for data reporting and connecting families and peers within pediatric-focused institutions. The second opinion article (Stasolla et al.) discussed the use of technology in the evaluation and recovery of post-coma and altered consciousness patients. In particular, the authors proposed the integration of assistive technology-based devices and virtual reality setups for both assessment and recovery purposes.

A mini-review (Wen et al.) examined the state of the art in Life Design Counseling (LDC), showing the lack of attention to clients from diverse backgrounds and professional counselors, the lack of different methods in the intervention process, and the lack of research on LDC. Abdel-Wahab et al. wrote a brief report to introduce clinical guidelines for the treatment of resistant depression in Egyptian patients. The guidelines were consistent with international recommendations and were the first in Egypt. However, the proposed guidelines had a limitation related to the fact that the panel consisted of only eight doctors.

The present Research Topic offers an overview of the latest experimental techniques and methods used to investigate fundamental issues in Psychology for Clinical Settings. The studies and opinion articles included in this Research Topic were conducted in different clinical populations and settings, which may represent a strength for the audience, researchers, and health professionals. Given the collected findings and the worldwide high prevalence of mental illness (World Health Organization, 2022a), continuous efforts to explore new clinical intervention and evaluation methodologies must be strongly encouraged. The latter is of special relevance after the impact of the pandemic (World Health Organization, 2022b).

## Author contributions

TC drafted the first version of this editorial. All authors contributed to and approved the final version.
